# Prognostic value of visual and quantitative CMR regional myocardial function in patients with suspected myocarditis

**DOI:** 10.1007/s10554-024-03059-1

**Published:** 2024-03-01

**Authors:** Benedikt Bernhard, Philippe Joss, Noah Greisser, Anselm W. Stark, Jonathan Schütze, Isaac Shiri, Yasaman Safarkhanlo, Kady Fischer, Dominik P. Guensch, Jessica A. M. Bastiaansen, Maryam Pavlicek, Dominik C. Benz, Raymond Y. Kwong, Christoph Gräni

**Affiliations:** 1 Cardiovascular Division, Department of Medicine, Brigham and Women’s Hospital, Harvard Medical School, Boston, MA USA; 2 Department of Cardiology, Inselspital, Bern University Hospital, University of Bern, Bern, Switzerland; 3 Department of Anaesthesiology and Pain Medicine, Inselspital, University Hospital Bern, University of Bern, Bern, Switzerland; 4 Department of Diagnostic, Interventional and Pediatric Radiology, Inselspital, Bern University Hospital, University of Bern, Bern, Switzerland; 5 Translation Imaging Center (TIC), Swiss Institute for Translational and Entrepreneurial Medicine, Bern, Switzerland; 6 Department of Cardiology, University Hospital Bern, Freiburgstrasse, CH – 3010 Bern, Switzerland

**Keywords:** CMR, Myocarditis, Regional strain, Global longitudinal strain, LLC

## Abstract

According to updated Lake-Louise Criteria, impaired regional myocardial function serves as a supportive criterion in diagnosing myocarditis. This study aimed to assess visual regional wall motional abnormalities (RWMA) and novel quantitative regional longitudinal peak strain (RLS) for risk stratification in the clinical setting of myocarditis. In patients undergoing CMR and meeting clinical criteria for suspected myocarditis global longitudinal strain (GLS), late gadolinium enhancement (LGE), RWMA and RLS were assessed in the anterior, septal, inferior, and lateral regions and correlated to the occurrence of major adverse cardiac events (MACE), including heart failure hospitalization, sustained ventricular tachycardia, recurrent myocarditis, and all-cause death. In 690 consecutive patients (age: 48.0 ± 16.0 years; 37.7% female) with suspected myocarditis impaired RLS was correlated with RWMA and LV-GLS but not with the presence of LGE. At median follow up of 3.8 years, MACE occurred in 116 (16.8%) patients. Both, RWMA and RLS in anterior-, septal-, inferior-, and lateral- locations were univariately associated with outcomes (all p < 0.001), but not after adjusting for clinical characteristics and LV-GLS. In the subgroup of patients with normal LV function, RWMA were not predictive of outcomes, whereas septal RLS had incremental and independent prognostic value over clinical characteristics (HR_adjusted_ = 1.132, 95% CI 1.020–1.256; p = 0.020). RWMA and RLS can be used to assess regional impairment of myocardial function in myocarditis but are of limited prognostic value in the overall population. However, in the subgroup of patients with normal LV function, septal RLS represents a distinctive marker of regional LV dysfunction, offering potential for risk-stratification.

**Graphical abstract:**

CI: confidence interval, CMR: cardiac magnetic resonance imaging, HR: hazard ratio, MACE major adverse cardiovascular events

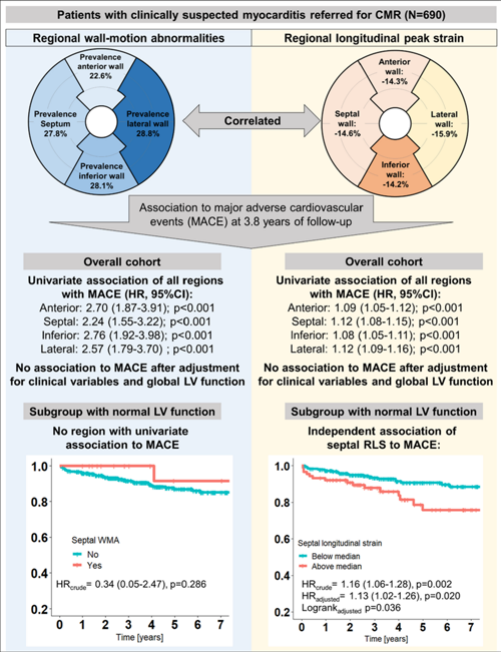

**Supplementary Information:**

The online version contains supplementary material available at 10.1007/s10554-024-03059-1.

## Introduction

Diagnosing myocarditis remains a challenge, due to its heterogeneous clinical presentation with a wide range of symptoms from asymptomatic cases to heart failure and sudden cardiac death [1, 2]. In the clinical setting of suspected myocarditis, cardiac magnetic resonance imaging (CMR) provides the ability to evaluate various functional and tissue characterization parameters in one comprehensive exam. The pivotal role of CMR to non-invasively diagnose myocarditis is underscored in the Lake Louise Criteria (LLC) [3]. In 2018 updated LLC encompass major criteria including non-ischemic myocardial injury evaluated by diffuse fibrosis and late gadolinium enhancement (LGE), and myocardial edema, while signs of pericarditis and global- as well as regional left ventricular (LV) dysfunction serve as supportive criteria [3]. For global LV function, the assessment of quantitative global longitudinal strain (GLS) by CMR feature tracking has proven to be of higher prognostic value over traditional functional assessments such as LV ejection fraction (EF) [4]. Currently, regional left ventricular (LV) dysfunction is mostly evaluated by qualitative visual assessment (i.e. visual regional wall motion abnormalities, RWMA). It is unclear whether RWMA or novel quantitative regional longitudinal peak strain (RLS) can help to risk stratify patients with suspected myocarditis. In this retrospective observational study, we sought to investigate the respective prognostic values of measurements of regional myocardial dysfunction from CMR (RWMA and RLS) for major adverse cardiovascular events (MACE) in suspected myocarditis. As myocarditis frequently affects localized regions, and patients present initially with normal global LV function, we also seek to determine if localized myocardial injury reflected by either RWMA or RLS is associated with adverse cardiac outcomes in patients with preserved global LV function.

## Methods

### Study design

Consecutive patients with clinical suspicion for myocarditis, who were referred for CMR, were included in two registries at tertiary centers (i.e. CMRMyo, CMR Features in Patients With Suspected Myocarditis registry, NCT03470571, and the FlamBer, Inflammatory Cardiomyopathy Bern Registry, NCT04774549). Patients were enrolled at the Brigham and Women’s Hospital, Harvard Medical School Boston, MA, USA between 2002 and 2015 and at Inselspital, University Hospital Bern, Switzerland between 2005 and 2019. Detailed inclusion and exclusion criteria for this cohort can be found elsewhere [4–8]. Patients who fulfilled clinical criteria for suspected myocarditis, as suggested by European Society of Cardiology (ESC) [1] were included in the final analysis. In short, ≥ 1 clinical criteria (e.g. acute chest pain, dyspnea) and ≥ 1 diagnostic criteria (e.g. abnormal ECG, elevated troponin) or presence of edema or LGE in a typical myocarditis pattern in CMR or ≥ 2 diagnostic criteria, were considered as clinically suspected myocarditis [1]. Follow up was performed at both centers by clinically indicated outpatient visits, standardized interviews, documentation from referring physicians and hospital discharge summaries. Endpoint was a composite of first MACE including all-cause death, hospitalization for congestive heart failure, documented sustained ventricular tachycardia for ≥ 30 s or recurrent myocarditis according to the ESC criteria [1]. The study was approved by the local ethics committees and was conducted in accordance with the Declaration of Helsinki.

### Image acquisition and analysis

CMR imaging was either performed on a 1.5 or 3.0 Tesla scanner (Magnetom Trio and Area, Siemens Healthineers, Erlangen, Germany) as described previously [4, 7, 9]. The application cvi41 (Circle Cardiovascular Imaging, Calgary, Canada) was used for postprocessing. Endo- and epicardial contours were automatically generated in end diastole and manually checked for plausibility in a short axis stack and three long axis (2-chamber, 3-chamber and 4-chamber view) cine sequences. Tracking these contours over the full cardiac cycle allowed to derive biventricular volumes, EF and feature tracking based peak global and RLS based on 16-segment American Heart Association (AHA) nomenclature [10]. RLS of each segment was individually checked for adequate time to peak and plausibility (e.g. only tracking during a part of the cardiac cycle). If required, values were either corrected for the peak strain at correct time to peak or excluded. Since reliability of segment based RLS is limited [9], segments were also summarized to different regions by calculating the mean RLS of summarized segments. Regions were defined as anterior (AHA-segments 1, 7, and 13), septal (segments 2, 3, 8, 9, 14), inferior (segments 4, 10, 15) and lateral (segments 5, 6, 11, 12, 16). RWMA were visually evaluated in a short-axis stack, according to regions depicted above. Patients with diffuse hypokinesis (LVEF < 30%) were categorized as having RWMA in each region. The LGE extent was semi-quantitatively determined by the sum of all AHA segments exhibiting LGE. For the sub-analysis of patients with normal LV function, LV-GLS below − 14.2% for women and below − 12.7% for men based on the publication by Kawel-Boehm et al. [11] was considered normal.

### Statistical analysis

Continuous variables were expressed as mean ± standard deviation or median and interquartile range based on normality. Categorical variables were presented as frequency and percent of the population. Patient characteristics and global findings are reported for the entire cohort, as well as for patients with and without normal global LV function, based on LV-GLS (see above). Groups were compared by a chi-square test or an independent t-test, for categorical or continuous variables, respectively. Pearson correlation was used to describe the association between RLS and GLS. Univariate and multivariate regression for the association with MACE was performed using Cox proportional hazards ratio (HR) and reported with 95% confidence intervals (CI). A multivariate Cox regression model for MACE was applied, including manually selected variables, which were not included in the diagnostic work up based on ESC criteria for suspected myocarditis [1]. Variables with > 10% missing values (e.g. results of parametric mapping) or those that could introduce multicollinearity (e.g. LV volumes in addition to LVEF) were not considered. The goodness of fit for each model was evaluated by Chi-Square from a likelihood ratio test and compared to the subsequent model for statistically significant differences. Kaplan Meier survival curves were plotted for data dichotomized by median septal RLS (< − 14.6% vs. ≥ 14.6%). Images of 25 randomly selected patients were replicated by the same reader and a second independent reader and inter‑ and intra-observer reliability of RWMA and RLS was assessed using a two-way intra-class correlation (ICC) test for absolute measures. ICC was interpreted according to Koo et al. (ICC ≥ 0.9–excellent, ICC ​ = ​0.75–0.89 – good, ICC ​ = ​0.5–0.74 – moderate, ICC < 0.5 – poor) [12]. Statistical significance was defined with a 2-sided p-value of < 0.05. Statistical analysis was performed with *R* software version 4.1.2. (R Foundation for Statistical Computing, Vienna Austria).

## Results

**Fig. 1:**
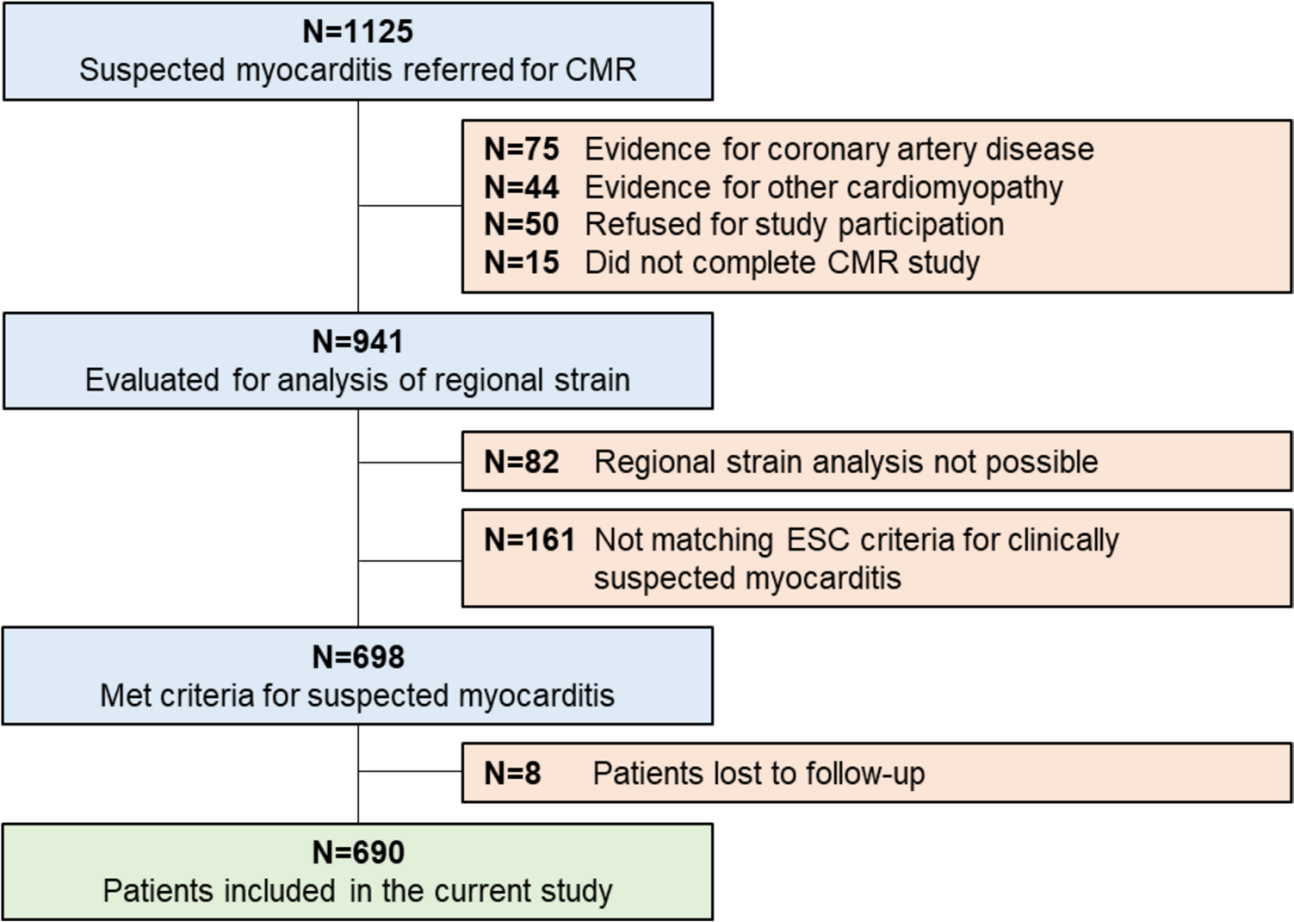
Study consort flow. *CMR* cardiac magnetic resonance, *ESC* European Society of Cardiology

**Fig. 2:**
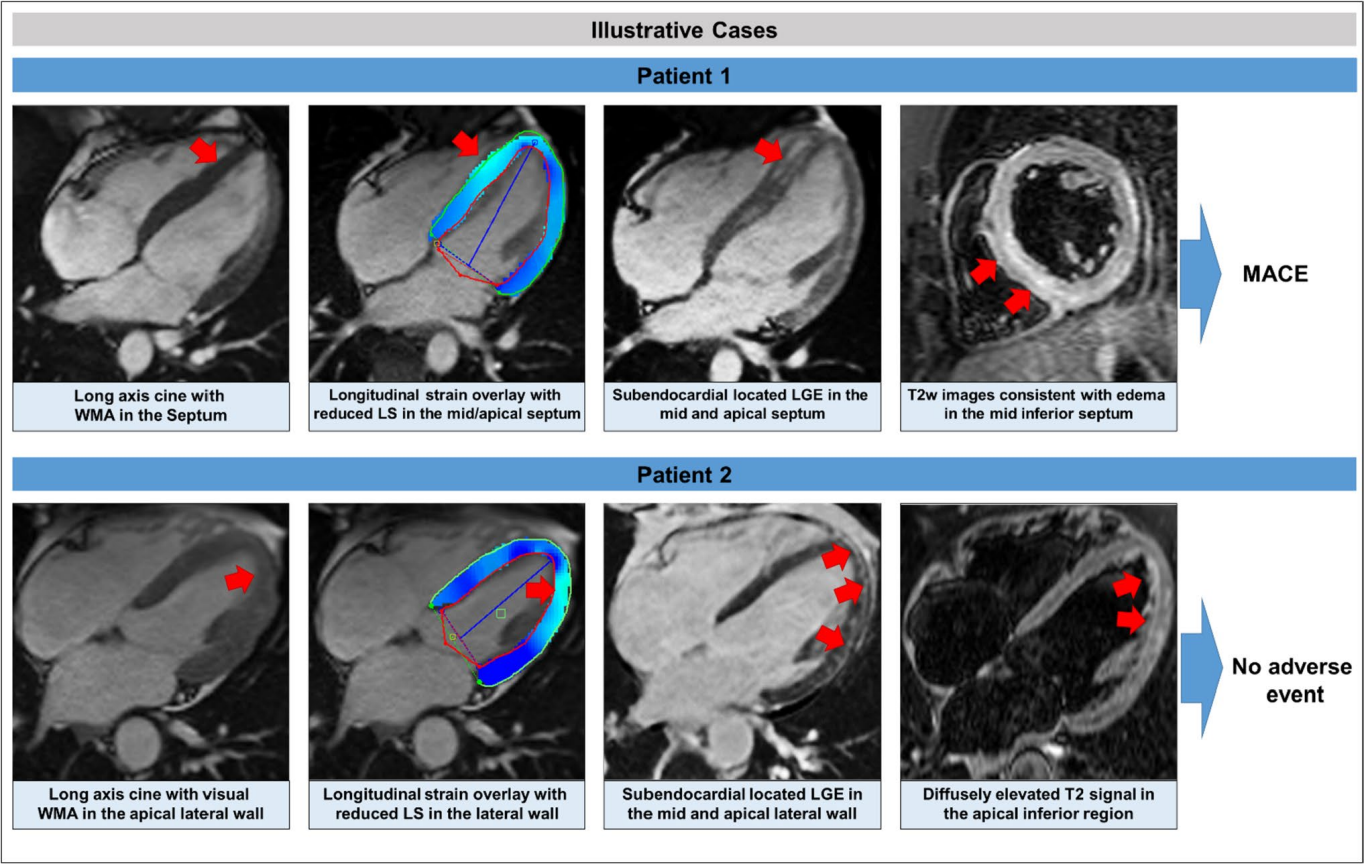
Illustrative cases. *LGE* late gadolinium enhancement, *LS* longitudinal strain, *MACE* major adverse cardiovascular events, *RWMA* regional wall motion abnormality

**Fig. 3:**
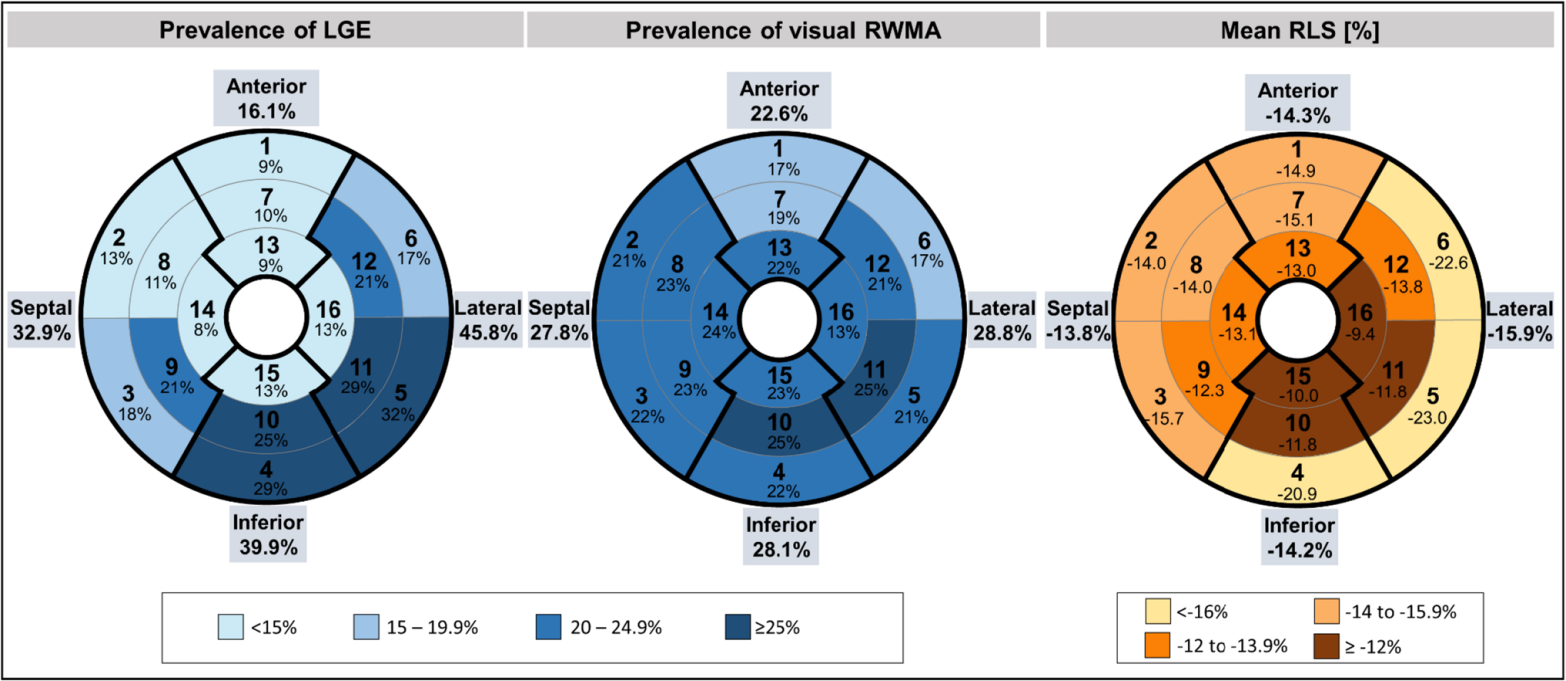
Prevalence of LGE and RWMA and mean RLS across regions. *LGE*- late gadolinium enhancement, *RLS* regional longitudinal peak strain, *RWMA* regional wall motion abnormalities

**Fig. 4:**
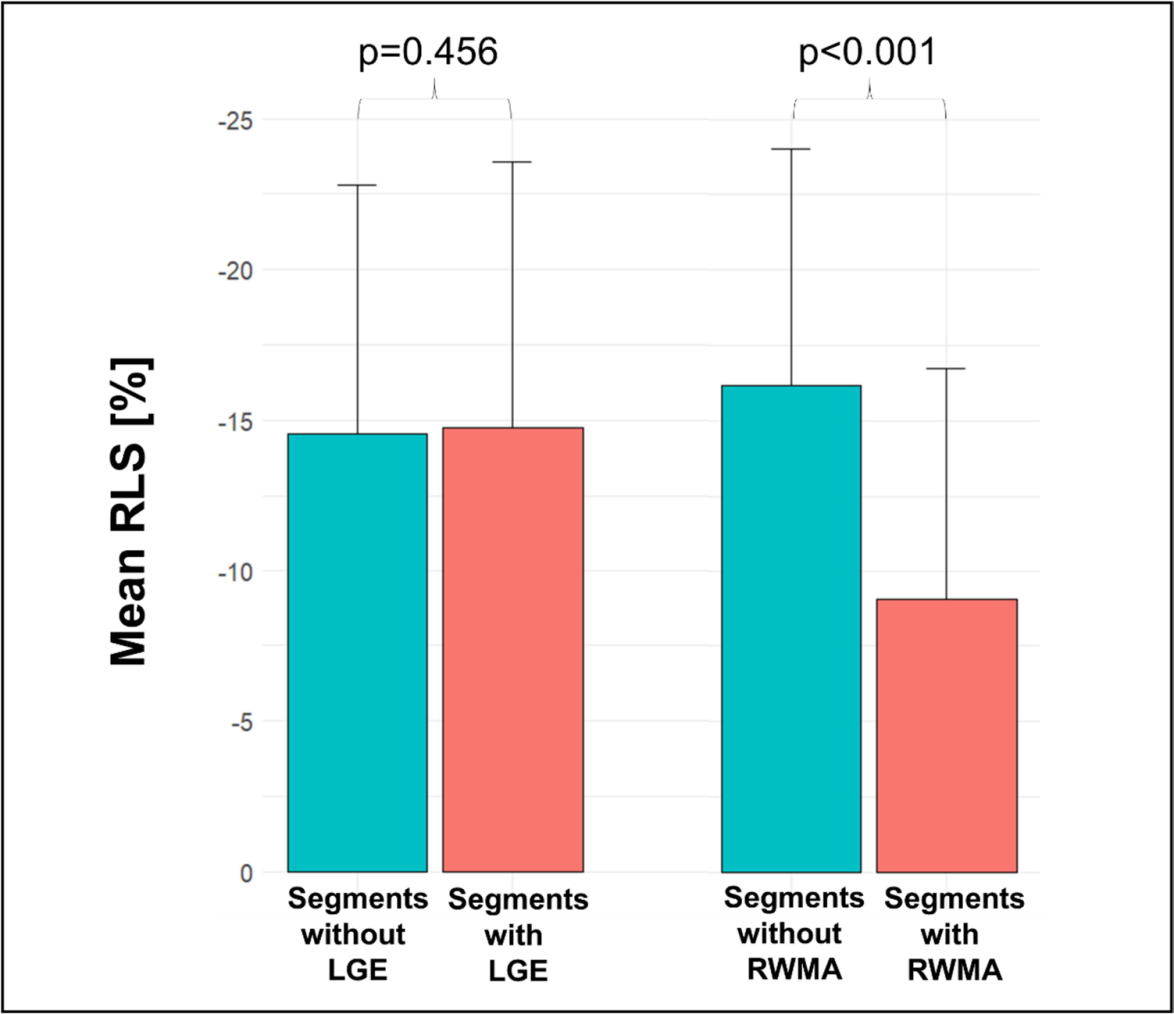
Mean RLS in regions with and without LGE and RWMA. LGE- late gadolinium enhancement, *RLS* regional longitudinal peak strain, *RWMA* regional wall motion abnormalities

Out of the 1,125 consecutive patients initially referred to CMR for suspected myocarditis, 75 (6.7%) were excluded due to evidence of coronary artery disease, 50 (4.4%) refused study participation, 44 (3.9%) suffered from other cardiomyopathies (e.g. cardiac amyloidosis, stress cardiomyopathy, hypertrophic cardiomyopathy, severe valvular heart disease, LV non-compaction) and 15 (1.3%) did not complete the CMR exam. Among the remaining 941 patients, in 82 (8.7%) cases regional strain analysis was not possible due to arrhythmia, missing or foreshortened 2-, 3-, or 4-chamber view or artefact. Another 161 (16.0%) patients did not meet ESC criteria for clinically suspected myocarditis (1) and 8 (1.1%) patients were lost to follow-up, leaving 690 patients for the current analysis (Fig. 1). Mean age was 48.0 ± 16.0 years and 260 (37.7%) were women (Table 1). Most common symptoms at admission were chest pain and dyspnea which were present in 259 (37.5%) and 300 (44.7%) patients, respectively. Common ECG alterations comprised T-wave inversions in 193 patients (30.4%), left bundle branch block in 55 (8.6%) and ST-segment elevation in 37 (5.4%) patients. Mean LVEF was 49.1 ± 15.0% and mean LV-GLS was -13.1 ± 4.4%. Global LV function, as defined by sex-specific cutoffs for LV-GLS mentioned above, was impaired in 303 (43.9%) patients (Table 2). LGE was present in 442 (64.1%) patients and edema in T2 weighted imaging or T2 mapping was observed in 211 (37.9%) of 557 patients without missing T2 based image sequences (Fig. 2). Regional analysis demonstrated high prevalence of LGE in the inferior and lateral region (i.e., AHA-segments 4, 5, 10 and 11) (Fig. 3). RWMA by visual assessment was most common (25%) in inferior and lateral regions and less common in the anterior segments. RLS across regions was associated with the presence of RWMA (p < 0.001) but not with the presence of LGE (p = 0.456) within that same region (Fig. 4). Mean RLS for each region with and without LGE or RWMA is displayed in Supplemental Fig. 1. RLS of all regions was correlated to LV-GLS with correlation coefficients ranging from 0.758 to 0.836 (Supplemental Fig. 2). Reproducibility of RLS was good to moderate and ICC for intra-reader reliability was between 0.84 and 0.93 and ranged from 0.70 to 0.89 for inter-reader reliability (Supplemental Table 1). Intra- and inter-reader agreement for assessment of RWMA was good and good to moderate, respectively (Supplemental Table 2).

**Table 1:** Patient characteristics

	Total population (n = 690)	Impaired global LV function (n = 303)	Normal global LV function (n = 387)	p-value
Patient characteristics				
Age [years]	48.0 ± 16.0	51.4 ± 14.4	45.4 ± 16.7	** < 0.001**
Sex (female)	260 (37.7%)	140 (46.2%)	120 (31.0%)	** < 0.001**
BMI [kg/m^2^]	27.3 ± 5.8	27.5 ± 6.3	27.2 ± 5.4	0.464
History of smoking	159 (23.0%)	62 (20.5%)	97 (25.1%)	0.182
History of Diabetes mellitus	58 (8.4%)	36 (11.9%)	22 (5.7%)	**0.006**
History of hypertension	188 (27.2%)	91 (30.0%)	97 (25.1%)	0.171
Hypercholesterinemia	147 (21.3%)	71 (23.4%)	76 (19.6%)	0.265
Symptoms at admission				
Chest pain	259 (37.5%)	69 (22.8%)	190 (49.1%)	** < 0.001**
Typical	124 (18.0%)	25 (8.3%)	99 (25.6%)	** < 0.001**
Atypical	135 (19.6%)	44 (14.5%)	91 (23.5%)	**0.004**
Arrhythmia	130 (18.8%)	47 (15.5%)	83 (21.4%)	0.060
Palpitations	43 (6.2%)	11 (3.6%)	32 (8.3%)	**0.019**
Syncope	44 (6.4%)	12 (4.0%)	32 (8.3%)	**0.032**
Cardiac arrest	27 (3.9%)	15 (5.0%)	12 (3.1%)	0.296
Dyspnea NYHA II- IV	300 (44.7%)	193 (65.6%)	107 (28.4%)	** < 0.001**
Biomarkers (peak median, IQR)				
Troponin [ng/l]*	20.0 (2–101)	4.5 (0–33)	46.0 (8–280)	** < 0.001**
Creatine-kinase [U/l]*	183 (77.5–503)	134 (63–339)	227 (93.5–568)	0.130
White blood cell count [*10^3^/ µl]*	8.2 (6.4–11.0)	8.5 (6.4–12.3)	7.9 (6.4–10.3)	**0.017**
ECG				
Absence of sinus rhythm	43 (6.8%)	24 (8.6%)	19 (5.4%)	0.153
Left bundle branch block	55 (8.6%)	44 (15.5%)	11 (3.1%)	** < 0.001**
ST-segment elevation	37 (5.4%)	4 (1.3%)	33 (8.5%)	** < 0.001**
ST-segment depression	14 (2.0%)	5 (1.7%)	9 (2.3%)	0.754
T wave inversion	193 (30.4%)	113 (39.9%)	80 (22.7%)	** < 0.001**

*BMI* body mass index, *LV* left ventricle, *NYHA* New York Heart Association

**Table 2:** CMR imaging characteristics

	Total population (n = 690)	Impaired global LV function (n = 303)	Normal global LV function (n = 387)	p-value
LV global function and dimensions				
LV EDV indexed [ml/m^2^]	97.9 ± 33.5	114 ± 41.2	85.5 ± 18.2	** < 0.001**
LV SV indexed [ml/m^2^]	53.3 ± 34.7	75.7 ± 41.3	36.1 ± 12.0	** < 0.001**
LV EF [%]	49.1 ± 15.0	37.3 ± 14.0	58.4 ± 7.2	** < 0.001**
LV GLS [%]	− 13.1 ± 4.4	− 9.0 ± 3.0	− 16.3 ± 2.0	** < 0.001**
LV GLS time to peak [ms]	321 ± 58.5	322 ± 66.2	320 ± 51.8	0.675
LV mass index [g/m^2^]	61.8 ± 16.5	67.9 ± 18.5	57.1 ± 12.9	** < 0.001**
Any WMA	265 (38.4%)	192 (63.4%)	73 (18.9%)	** < 0.001**
LV regional function				
Anterior RLS [%]	− 14.3 ± 5.3	− 10.5 ± 4.6	− 17.2 ± 3.7	** < 0.001**
Septal RLS [%]	− 13.8 ± 4.9	− 10.1 ± 4.4	− 16.7 ± 3.0	** < 0.001**
Inferior RLS [%]	− 14.2 ± 5.5	− 10.2 ± 4.5	− 17.4 ± 4.0	** < 0.001**
Lateral RLS [%]	− 15.9 ± 5.2	− 12.1 ± 4.4	− 18.9 ± 3.6	** < 0.001**
Anterior WMA	155 (22.5%)	143 (47.2%)	12 (3.1%)	** < 0.001**
Septal WMA	191 (27.7%)	167 (55.1%)	24 (6.2%)	** < 0.001**
Inferior WMA	193 (28.0%)	153 (50.5%)	40 (10.3%)	** < 0.001**
Lateral WMA	198 (28.7%)	150 (49.5%)	48 (12.4%)	** < 0.001**
LV global tissue characteristics				
LGE presence	442 (64.1%)	192 (63.4%)	250 (64.6%)	0.799
LGE score [segments]	2.8 ± 3.2	2.9 ± 3.5	2.7 ± 2.9	0.462
Native T1 at 1.5 T [ms]*	1020 ± 40.8	1030 ± 42.1	1010 ± 39.0	0.281
Native T1 at 3 T [ms]*	1130 ± 41.6	1130 ± 43.8	1140 ± 39.1	0.844
ECV [%]*	31.9 ± 6.01	32.0 ± 5.6	31.8 ± 6.5	0.807
Myocardial edema by T2*	211 (37.9%)	97 (41.5%)	114 (35.3%)	0.164
LV regional tissue characteristics				
Anterior LGE	111 (16.1%)	55 (18.2%)	56 (14.5%)	0.229
Septal LGE	227 (32.9%)	134 (44.2%)	93 (24.0%)	** < 0.001**
Inferior LGE	275 (39.9%)	106 (35.0%)	169 (43.7%)	**0.026**
Lateral LGE	316 (45.8%)	117 (38.6%)	199 (51.4%)	**0.001**

*ECV* extracellular volume fraction, *EDV* end diastolic volume, *EF* ejection fraction, *GLS* global longitudinal strain, *LGE* late gadolinium enhancement, *LV* left ventricle, *RLS* regional longitudinal peak strain, *SV* stroke volume, *RWMA* regional wall motion abnormalities

*Variables with > 10% missing values

At median follow up of 3.8 years, MACE occurred in 116 (16.8%) patients, including heart failure hospitalization (40; 5.8%), sustained ventricular tachycardia (29; 4.2%), recurrent myocarditis (18; 2.6%), and all-cause death (29; 4.2%). MACE stratified by LVEF is presented in Supplemental Table 3. In addition to parameters of global LV function such as LVEF, LV-GLS and the presence of visually assessed RWMA, LGE and clinical characteristics including body-mass index (BMI), smoking, and a history of diabetes were associated with MACE in the univariate analysis. Anterior, septal, inferior, and lateral RLS and RWMA were also univariately associated with outcomes, while septal LGE was the only regional LGE pattern associated with MACE (Table 3). A basic multivariable predictive model was defined based on the results of univariate cox-regression and included BMI, smoking, history of diabetes mellitus, LVEF, LV-GLS, and LGE extent (Model χ2 = 65.86). None of the regional findings (RLS, RWMA and LGE) sequentially added to this model improved prognostication (Table 4) for the overall cohort. Separate results after removing LV GLS and LVEF from the multivariable models are provided in Supplemental Table 4.

**Table 3:** Cox-Regression Model—Univariate association with MACE

	Total population (n = 690, 116 with event)		Impaired global LV function (n = 303, 77 with event)		Normal global LV function (n = 387, 39 with event)	
	HR (95%CI)	p	HR (95%CI)	p	HR (95%CI)	p
Patient characteristics						
Age [years]	1.011 (0.999–1.022)	0.073	1.006 (0.991–1.023)	0.426	1.005 (0.986–1.024)	0.619
Sex (female)	1.366 (0.948–1.970)	0.095	1.474 (0.940–2.313)	0.091	0.671 (0.318–1.414)	0.294
BMI [kg/m^2^]	1.045 (1.015–1.076)	**0.003**	1.020 (0.984–1.057)	0.283	1.091 (1.039–1.145)	** < 0.001**
History of smoking	1.920 (1.312–2.810)	**0.001**	1.463 (0.879–2.436)	0.143	3.537 (1.883–6.643)	** < 0.001**
History of diabetes mellitus	1.797 (1.044–3.093)	**0.034**	1.470 (0.793–2.723)	0.221	1.521 (0.468–4.948)	0.485
History of hypertension	1.314 (0.886–1.949)	0.174	1.032 (0.628–1.695)	0.902	1.800 (0.934–3.469)	0.079
Hypercholesterinemia	1.052 (0.661–1.675)	0.830	1.148 (0.652–2.020)	0.632	1.010 (0.446–2.291)	0.981
LV global function and dimensions						
LV EDV indexed [ml/m^2^]	1.010 (1.005–1.014)	** < 0.001**	1.006 (1.000–1.011)	**0.038**	1.001 (0.984–1.018)	0.912
LV SV indexed [ml/m^2^]	1.011 (1.007–1.015)	** < 0.001**	1.007 (1.002–1.012)	**0.007**	1.009 (0.985–1.034)	0.479
LV EF [%]	0.962 (0.951–0.972)	** < 0.001**	0.966 (0.950–0.983)	** < 0.001**	0.974 (0.935–1.015)	0.217
LV-GLS [%]	1.152 (1.107–1.200)	** < 0.001**	NA	NA	NA	NA
LV mass index [g/m^2^]	1.008 (0.997–1.020)	0.133	0.997 (0.984–1.010)	0.643	1.010 (0.986–1.034)	0.421
Any RWMA	2.048 (1.417–2.960)	** < 0.001**	1.503 (0.902–2.507)	0.118	1.110 (0.526–2.344)	0.784
LV regional function						
Anterior RLS [%]	1.086 (1.053–1.120)	** < 0.001**	1.061 (1.014–1.109)	**0.011**	1.022 (0.942–1.108)	0.605
Septal RLS [%]	1.116 (1.081–1.153)	** < 0.001**	1.071 (1.021–1.124)	**0.005**	1.162 (1.058–1.277)	**0.002**
Inferior RLS [%]	1.078 (1.047–1.111)	** < 0.001**	1.068 (1.021–1.117)	**0.004**	0.964 (0.891–1.043)	0.365
Lateral RLS [%]	1.124 (1.087–1.162)	** < 0.001**	1.107 (1.052–1.164)	** < 0.001**	1.080 (0.998–1.169)	0.055
Anterior RWMA	2.702 (1.869–3.906)	** < 0.001**	1.762 (1.114–2.787)	**0.016**	1.715 (0.413–7.125)	0.458
Septal RWMA	2.236 (1.552–3.223)	** < 0.001**	1.661 (1.030–2.677)	**0.037**	1.339 (0.947–2.474)	0.286
Inferior RWMA	2.765 (1.920–3.981)	** < 0.001**	2.267 (1.390–3.697)	**0.001**	1.354 (0.567–3.235)	0.495
Lateral RWMA	2.573 (1.788–3.703)	** < 0.001**	2.029 (1.264–3.256)	**0.003**	1.461 (0.645–3.311)	0.364
LV global tissue characteristics						
LGE	1.558 (1.036–2.343)	**0.033**	1.592 (0.968–2.618)	0.067	1.657 (0.807–3.402)	0.169
LGE extent [No. of segments]	1.081 (1.028–1.137)	**0.002**	1.079 (1.022–1.14)	**0.007**	1.056 (0.955–1.167)	0.292
LV regional tissue characteristics						
Anterior LGE	1.521 (0.983–2.354)	0.060	1.531 (0.912–2.570)	0.107	1.237 (0.546–2.804)	0.611
Septal LGE	1.824 (1.264–2.633)	**0.001**	1.636 (1.042–2.567)	**0.032**	1.260 (0.627–2.533)	0.516
Inferior LGE	1.381 (0.958–1.991)	0.084	1.774 (1.129–2.788)	**0.013**	1.237 (0.659–2.321)	0.507
Lateral LGE	1.032 (0.716–1.488)	0.866	1.185 (0.753–1.865)	0.463	1.168 (0.620–2.199)	0.630

*BMI* body mass index, *EDV* end diastolic volume, *EF* ejection fraction, *GLS* global longitudinal strain, *LGE* late gadolinium enhancement, *LV* left ventricle, *RLS* regional longitudinal peak strain, *SV* stroke volume, RWMA regional wall motion abnormalities

**Table 4:** Cox-Regression Model—Multivariate association with MACE

Variable selection	Total population (n = 690, 116 with event)		Impaired global LV function (n = 303, 77 with event)		Normal global LV function (n = 387, 39 with event)	
Basic model						
Variables (based on univariate analysis)	BMI, Smoking, Diabetes mellitus, LV EF, LV GLS, LGE extent		LV EF, LGE extent		BMI, Smoking	
Model χ2	65.86	p < 0.001	19.25	p < 0.001	21.87	p < 0.001

Sequentially added regional findings	Model χ^2^	p vs. basic model	Model χ^2^	p vs. basic model	Model χ^2^	p vs. basic model
Anterior RLS [%]	63.04	0.732	19.32	0.793	22.23	0.545
Septal RLS [%]	67.01	0.284	19.57	0.573	27.40	**0.019**
Inferior RLS [%]	67.11	0.265	19.54	0.593	22.56	0.405
Lateral RLS [%]	68.38	0.113	21.94	0.101	22.73	0.354
Anterior RWMA	65.86	0.987	19.45	0.654	25.00	0.077
Septal RWMA	67.45	0.208	19.96	0.399	22.60	0.393
Inferior RWMA	66.69	0.363	20.44	0.275	22.98	0.292
Lateral RWMA	66.27	0.523	19.62	0.544	23.47	0.205
Septal LGE	65.86	0.956	19.27	0.887	22.39	0.470
Inferior LGE	67.73	0.092	23.05	0.051	21.93	0.805

Final models	HR (95%CI)	p	HR (95%CI)	p	HR (95%CI)	p
Model χ2	65.86	** < 0.001**	19.25	** < 0.001**	28.16	** < 0.001**
BMI [kg/m^2^]	1.033 (1.002–1.065)	**0.035**			1.072 (1.018–1.129)	**0.008**
History of smoking	1.853 (1.261–2.724)	**0.002**			3.050 (1.621–5.740)	**0.001**
History of diabetes mellitus	1.292 (0.726–2.299)	0.383				
LV EF [%]	0.985 (0.962–1.008)	0.194	0.970 (0.953–0.986)	** < 0.001**		
LV GLS [%]	1.090 (1.002–1.185)	**0.046**				
LGE extent [No. of segments]	1.064 (1.011–1.119)	**0.018**	1.061 (1.003–1.122)	**0.038**		
Septal RLS [%]					1.132 (1.02–1.256)	**0.020**

*BMI* body mass index, *EDV* end diastolic volume, *EF* ejection fraction, *GLS* global longitudinal strain, *LGE* late gadolinium enhancement, *LV* left ventricle, *RLS* regional longitudinal peak strain, *SV* stroke volume, *RWMA* regional wall motion abnormalities

**Fig. 5:**
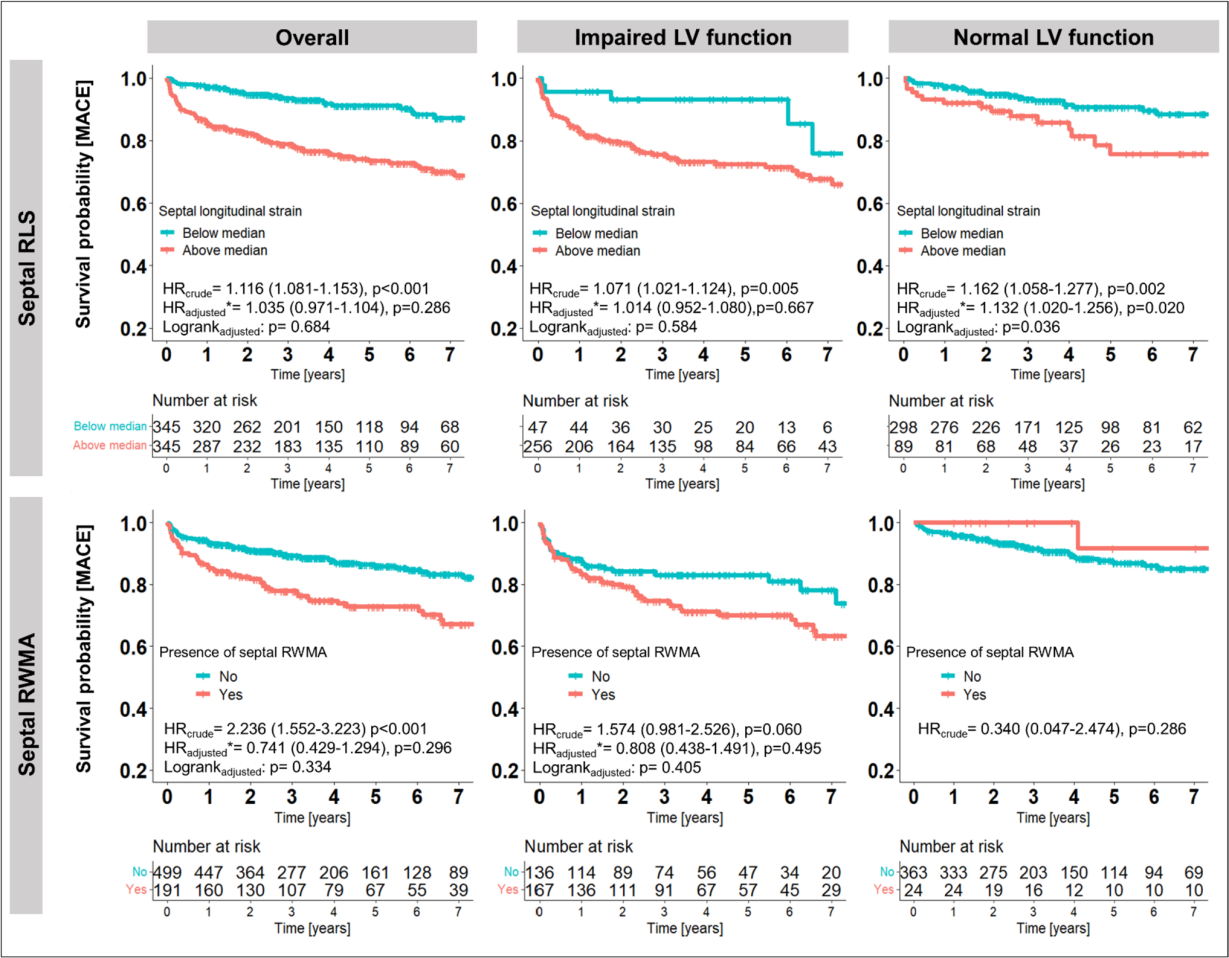
Kaplan Meier curves stratified by median septal RLS (-14.6%) and the presence of visually assessed RWMA for the composite endpoint of MACE. *Hazard ratios and log-rank tests for the overall cohort were adjusted for body-mass-index, history of smoking and diabetes, LVEF, LV GLS and LGE extent. Hazard ratios and log rank tests for the subgroup of patients with impaired LV function were adjusted for LVEF and LGE extent. Hazard ratios and log rank tests for the subgroup of patients with normal LV function were adjusted for body-mass index and history of smoking. *LGE* late gadolinium enhancement, *MACE* major adverse cardiac events, *RLS* regional longitudinal peak strain, *RWMA* wall motion abnormalities

For sub analyses, patients were stratified by LV-GLS into those with normal and impaired global LV function. Those with normal LV function were younger (p = 0.001), more often male (p < 0.001), less commonly suffered from diabetes mellitus (p = 0.006) and had more often chest pain symptoms (p < 0.001) but less dyspnea (p < 0.001) at admission (Table 1). LV function by LVEF, RLS and presence of RWMA correlated strongly to LV-GLS (Table 2). In patients with impaired LV function, several imaging parameters, including LVEF, RLS and RWMA of all regions, and LGE extent were associated with MACE, while in patients with normal LV function only clinical characteristics (BMI and smoking) and septal RLS were associated with outcomes (Table 3). Basic models incorporated LVEF and LGE extent in patients with impaired LV function, while BMI and smoking were included in the basic model for patients with normal LV function. The addition of regional findings demonstrated incremental and independent prognostic value of septal RLS over clinical characteristics in patients with normal LV function (HR_adjusted_ = 1.132, 95% CI 1.020–1.256; p = 0.002), while no independent effect was observed in those with impaired LV function (Fig. 5). Visually assessed RWMA were not associated with outcomes in this subgroup of patients (Graphical Abstract).

## Discussion

This study for the first time investigated the prognostic value of visual and quantitative regional myocardial dysfunction in suspected myocarditis. Both the presence of RWMA and RLS had moderate to high measurement reproducibility and were associated with clinical outcomes. However, in the model adjusted to key clinical risk markers, regional LV dysfunction, indicated by either RLS or RWMA, did not independently predict clinical outcomes, thus is of limited predictive value in the overall cohort of patients with suspected myocarditis. In the subgroup of patients with preserved LV function septal RLS demonstrated incremental and independent association with MACE. Impaired septal RLS might represent a high-risk pattern and incorporation into risk stratification may refine the prediction of MACE in this subgroup.

RLS has been demonstrated to be useful in the detection of RWMA in patients with ischemic heart disease [13–15], to be correlated with LGE [14–16], and inherits predictive value in patients following myocardial infarction and chronic total occlusion [17, 18]. Our hypothesis was that also in suspected myocarditis impaired RLS and RWMA might indicate areas of regional inflammation or scarring. However, contrasting the findings made in ischemic heart disease, RLS and RWMA were neither associated with regional LGE nor had independent prognostic value over global LV function. Following myocardial infarction, myocardial scarring is more often transmural and limited to a specific area of coronary supply, while in myocarditis less dense subendocardial or patchy LGE pattern are common [7, 19], that are notably less strong associated to impaired regional function. Consequently, RLS and RWMA were strongly associated to LV-GLS in our study, which might explain the lack of incremental value over LV-GLS. Even if of limited prognostic value, RLS is a reproducible tool and correlated well to visually assessed RWMA, suggesting a diagnostic role as supportive criterion outlined in the updated LLC in patients with suspected myocarditis [3].

Consistent with our results, recent studies have shown the prognostic potential of several CMR parameters including LGE, LVEF and GLS in suspected myocarditis [4–7, 20–23]. However, in patients with normal global LV function, these parameters are of limited value and do not incrementally and independently predict outcomes. Although patients with normal LV function are at significantly lower risk for adverse events, a certain proportion of these patients still develops heart failure, ventricular tachycardia, or recurrent myocarditis at follow-up, underlying the need for better risk stratification in this subgroup. Impairment of septal RLS was associated with outcomes in this patient population, which is in line with previous studies, that identified septal involvement by LGE as a high-risk feature in myocarditis [7, 19] or ischemic heart disease [24]. Septal involvement might indicate extending of inflammation to the right ventricle or can go along with involvement of the conduction system, both of which are associated with poor outcomes [8, 25–27]. In fact, whereas septal LGE was a strong outcome predictor in our sub-cohort with impaired LV function, septal LGE was not associated with outcomes in the subcohort with preserved LV function. Therefore, and in addition to widely established tissue characterization by LGE, septal RLS might represent a marker for septal involvement and may help to refine risk stratification especially in patients with preserved global LV function.

So far, RLS has not yet been implemented in clinical practice and is rather seen as a research tool. The application and interpretation of RLS is challenging and several limitations of the technique should be considered. First, RLS shows limited inter-reader reproducibility when assessed on basis of AHA-segmentation [28, 29], emphasizing the need to cluster segments to regions, as done by this study and to standardize the post-processing. However, a definition of which AHA-segments should be included to a region is rather arbitrary, especially in diseases like myocarditis without a coronary-anatomy-based distribution of lesions. Additionally, through a tethering of strain, regions with normal myocardium neighboring impaired segments can also exhibit low RLS [14, 30, 31]. Finally, no reference values exist for different RLS pattern, which is further complicated by inter-vendor variabilities and limited comparability to echocardiography. Addressing these aspects would allow for a more widespread use of RLS not only in suspected myocarditis, but also in other cardiac disease where regional differences in myocardial function are expected.

## Limitations

Several limitations require attention. First, this was a retrospective study, and the heterogeneous nature of myocarditis presentations makes the patient selection prone to selection bias. We tried to mitigate this concern by using standardized inclusion criteria for clinically suspected myocarditis according to the published ESC recommendations and by excluding all other cardiomyopathies and ischemic heart disease. Second, parametric mapping sequences (T1, T2 and extracellular volume fraction) were only available in more recently scanned patients and were therefore not included to outcome analysis or correlated to RLS. Third, since no reference values are available for regional peak strain assessed by CMR, cut-offs for Kaplan Meier curves were selected based on median values in our cohort. Fifths, follow-up imaging, such as repeat CMR to detect dilative cardiomyopathy as a consequence of acute myocarditis was not available for the majority of study participants. Sixths, although based on our experience we believe that the learning curve for the assessment of regional strain peaks early if a standardized protocol is followed, we did not analyze reproducibility over time. Finally, we did not confirm the diagnosis of myocarditis by a diagnostic gold-standard such as endomyocardial biopsy, which reflect current practice. Further, endomyocardial biopsy is prone to sampling error, associated with a non-negligible risk, and is currently reserved for more severe cases.

## Conclusion

RLS is a feasible tool to assess regional impairment of myocardial function and correlates to visually assessed RWMA. Both are associated with outcomes but are of lower prognostic value when compared to LV GLS. Nevertheless, in the subgroup of patients with normal LV function, septal RLS represents a marker of regional LV dysfunction with higher prognostic value than RWMA and can be used to refine risk-stratification in suspected myocarditis.

### Supplementary Information

Below is the link to the electronic supplementary material.

Supplementary file1 (DOCX 601 KB)

## Data Availability

Data available upon reasonable request from the corresponding author.

## Abbreviations

AHA: American Heart Association
CAD: Coronary artery disease
CI: Confidence Interval
CMR: Cardiovascular Magnetic Resonance Imaging
ECV: Extracellular volume
EMB: Endomyocardial biopsy
ESC: European society of cardiology
FWHM: Full width half maximum
GLS: Global longitudinal peak strain
HR: Hazard ratio
ICC: Intra-class correlation coefficient
LGE: Late gadolinium enhancement
LLC: Lake Louise criteria
LVEF: Left ventricular ejection fraction
MACE: Major adverse cardiac events
RLS: Regional longitudinal peak strain
RWMA: Visual regional wall motional abnormalities
