# Supplementary material for: Prognostic value of visual and quantitative CMR regional myocardial function in patients with suspected myocarditis
Source: Int J Cardiovasc Imaging. 2024 Mar 1;40(4):907–20. doi: 10.1007/s10554-024-03059-1 (PMC11052711; doi:10.1007/s10554-024-03059-1)

## Supplemental Material

**Supplemental Table 1: Reproducibility of regional longitudinal peak strain in 25 randomly selected patients**

|  | **Intra-reader reliability** | **Inter-reader reliability** |
| --- | --- | --- |
|  | **ICC (95% CI)** | **ICC (95% CI)** |
| Anterior RLS [%] | 0.845 (0.606-0.937) | 0.701 (0.242-0.882) |
| Septal RLS [%] | 0.855 (0.674-0.936) | 0.722 (0.358-0.880) |
| Inferior RLS [%] | 0.850 (0.630-0.939) | 0.886 (0.725-0.954) |
| Lateral RLS [%] | 0.928 (0.830-0.969) | 0.799 (0.543-0.912) |

**Abbreviation**s: ICC- Intra-class correlation coefficient; RLS - regional longitudinal peak strain

**Supplemental Table 2: Reproducibility of visually assessed regional wall-motion abnormalities in 25 randomly selected patients**

|  | **Intra-reader agreement** | **Inter-reader agreement** |
| --- | --- | --- |
| **Any RWMA** | 23/25 (92%) | 20/25 (80%) |
| Anterior RWMA | 24/25 (96%) | 21/25 (84%) |
| Septal RWMA | 23/25 (92%) | 20/25 (80%) |
| Inferior RWMA | 24/25 (96%) | 23/25 (92%) |
| Lateral RWMA | 25/25 (100%) | 24/25 (96%) |

**Abbreviation**s: RWMA – regional wall motion abnormalities

**Supplemental Table 3: MACE and MACE types according to LV function**

|  | **LVEF >50% N=394** | **LVEF ≤50% and >30% N=138** | **LVEF ≤30% N=113** |
| --- | --- | --- | --- |
| Any MACE | 44 (11.1%) | 21 (15.2%) | 51 (45.1%) |
| HF Hospitalization | 6 (1.5%) | 6 (4.3%) | 28 (24.8%) |
| Ventricular Tachycardia | 10 (2.5%) | 8 (5.8%) | 11 (9.7%) |
| Recurrent Myocarditis | 16 (4.1%) | 2 (1.4%) | 0 (0%) |
| Death | 12 (3.0%) | 5 (3.6) | 12 (10.6%) |

**Abbreviations:** HF – heart failure, LVEF – left ventricular ejection fraction, MACE – major adverse cardiovascular events,

**Supplemental Table 4: Multivariable models for regional findings after removing LV GLS and LVEF**

| **Variable selection** | **Model without LV GLS** (n=690, 116 with event) | | **Model without LVEF** (n=690, 116 with event) | |
| --- | --- | --- | --- | --- |
| **Basic Model** |  |  |  |  |
| Variables (based on  univariate analysis) | BMI, Smoking, Diabetes mellitus, LV EF, LGE extent | | BMI, Smoking, Diabetes mellitus, LV GLS, LGE extent | |
| Model χ2 | 62.9 | p<0.001 | 64.1 | p<0.001 |
| **Sequentially added regional findings** | **Model χ^2^** | **p vs. basic model** | **Model χ^2^** | **p vs. basic model** |
| Anterior RLS [%] | 63.2 | 0.596 | 64.2 | 0.806 |
| Septal RLS [%] | 68.3 | **0.049** | 65.7 | 0.215 |
| Inferior RLS [%] | 62.9 | 0.985 | 65.1 | 0.244 |
| Lateral RLS [%] | 70.2 | **0.017** | 67.0 | 0.093 |
| Anterior RWMA | 63.0 | 0.705 | 64.4 | 0.649 |
| Septal RWMA | 63.7 | 0.352 | 64.7 | 0.468 |
| Inferior RWMA | 64.0 | 0.289 | 65.9 | 0.191 |
| Lateral RWMA | 63.4 | 0.476 | 65.2 | 0.293 |

**Abbreviations**: BMI – body mass index, EF – ejection fraction, GLS – global longitudinal strain, LGE – late gadolinium enhancement, LV – left ventricle, RLS – regional longitudinal peak strain, RWMA – regional wall motion abnormalities

**Supplemental Figure 1: Mean regional longitudinal peak strain in regions with and without LGE and RWMA**

**
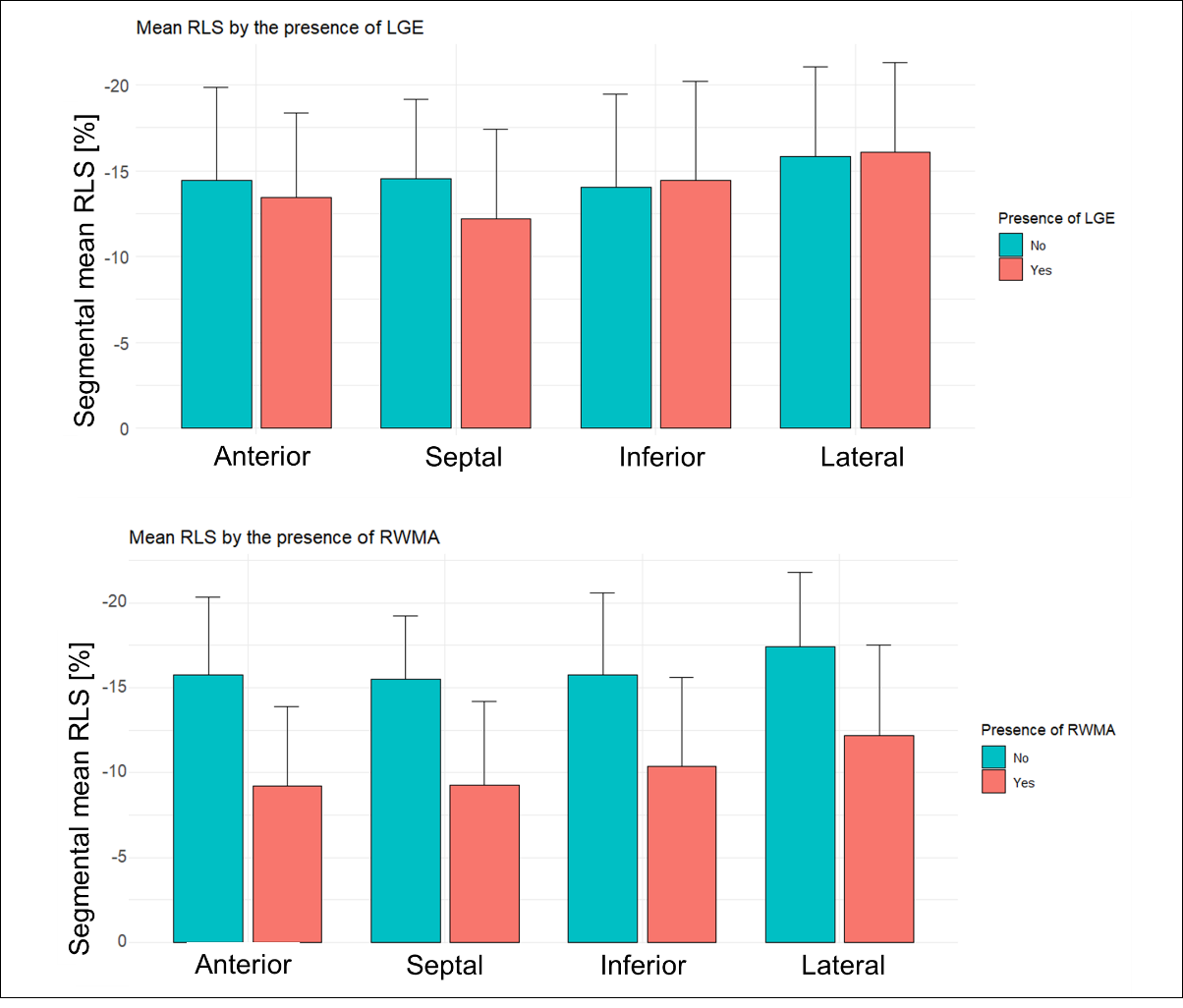
**

Abbreviations: AHA – American Heart Association, LGE – late gadolinium enhancement, RLS – regional longitudinal peak strain, RWMA – regional wall motion abnormalities

**Supplemental Figure 2: Correlation of global and regional longitudinal strain**


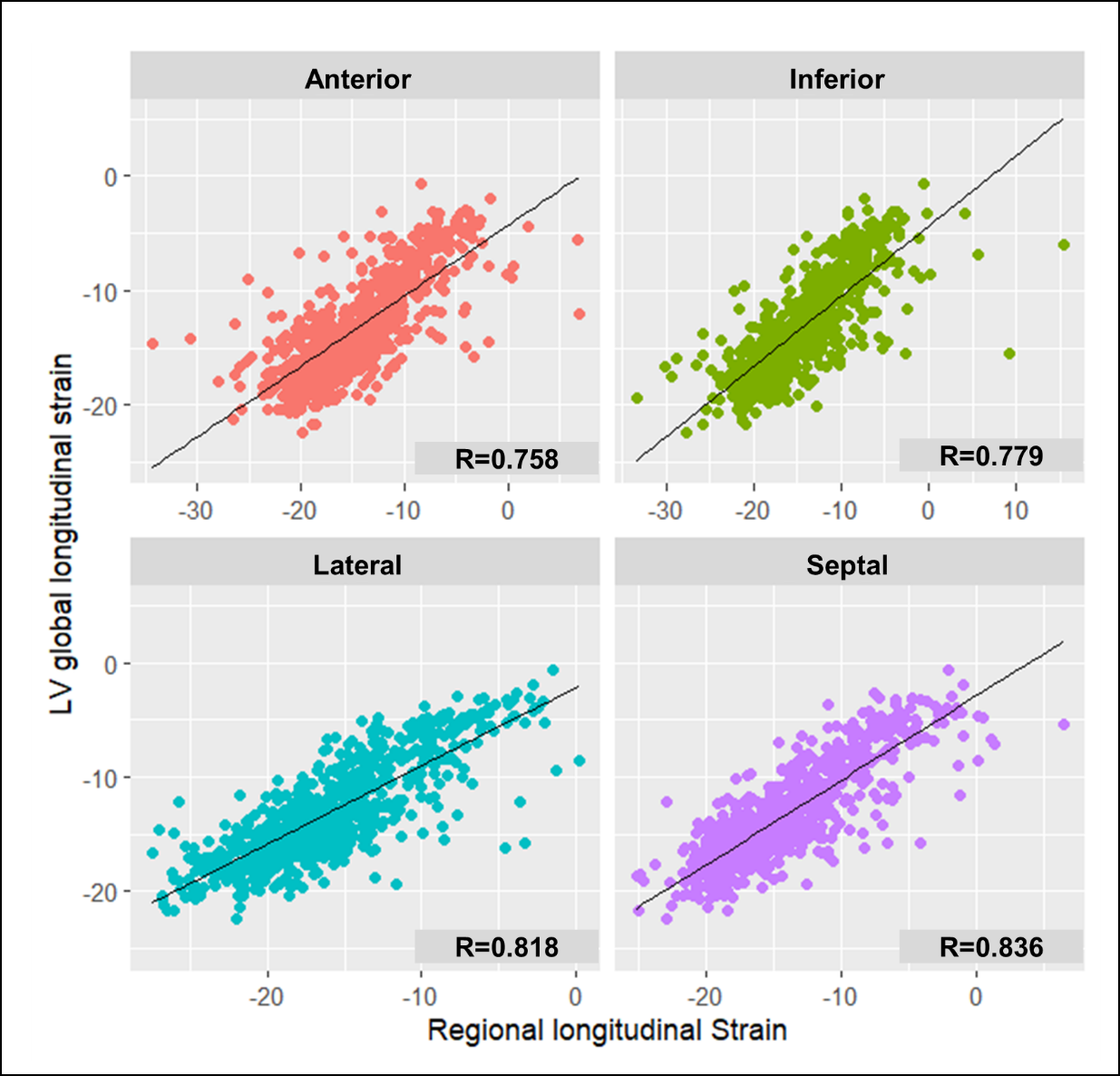

Supplement: Supplementary file 1 — Supplementary file1 (DOCX 601 KB) [file 10554_2024_3059_MOESM1_ESM.docx]
